# A risk model based on pyroptosis subtypes predicts tumor immune microenvironment and guides chemotherapy and immunotherapy in bladder cancer

**DOI:** 10.1038/s41598-022-26110-4

**Published:** 2022-12-12

**Authors:** Tielin Wu, Sheng Li, Chao Yu, Yuanbo Wu, Huimin Long

**Affiliations:** Department of Urology, Ningbo Medical Center Li Huili Hospital, 315199 Ningbo, China

**Keywords:** Cancer, Computational biology and bioinformatics, Immunology, Urology

## Abstract

Although immunotherapy has revolutionized bladder cancer (BLCA) therapy, only few patients demonstrate durable clinical benefits due to the heterogeneity. Emerging evidence has linked pyroptosis to shaping tumor microenvironment (TME) and predicting therapy response. However, the relationship between pyroptosis and immunotherapy response in BLCA remains elusive. In this study, we performed a comprehensive bioinformatic analysis to dissect the role of pyroptosis in BLCA. Differentially expressed pyroptosis-related genes (DEPRGs) between tumor and normal tissues were identified using publicly available datasets. Kaplan–Meier analysis was performed to screen for DEPRGs associated with survival. Consensus clustering was used for BLCA subtyping. TME characteristics were evaluated by CIBERSORT, ESTIMATE and immune checkpoint genes (ICGs). Following univariate COX regression and LASSO analyses with pyroptosis-related DEGs, the risk model and nomogram were constructed with TCGA dataset and validated in the GEO dataset. Furthermore, therapeutic responses in high- and low-risk groups were compared using TIDE and GDSC databases. Two pyroptosis-related subtypes (Cluster 1 and 2) were identified based on expression patterns of *GSDMA* and *CHMP4C*. Bioinformatic analyses showed that cluster 1 had poor survival, more M0/M1/M2 macrophages, higher immune/stromal/ESTIMATE scores, and higher expression levels of ICGs. A 15-gene signature for predicting prognosis could classify patients into high- and low-risk groups. Furthermore, the correlation of risk scores with TIDE score and IC_50_ showed that patients in low-risk group were more sensitive to immunotherapy, whereas patients in high-risk group could better benefit from chemotherapy. Our study identified two novel pyroptosis-related subtypes and constructed a risk model, which can predict the prognosis, improve our understanding the role of PRGs in BLCA, and guide chemotherapy and immunotherapy.

## Introduction

Despite the substantial advances in the surgical and systemic treatments over the past few decades, bladder cancer (BLCA) remains one of the most commonly diagnosed cancers with high morbidity and mortality rates worldwide. Globally, an estimated 573,278 new BLCA cases and 212,536 cancer deaths occurred in 2020^1^. BLCA is characterized by high-level genomic instability and heterogeneity, resulting in the formation of at least 40 histological subgroups^2^. The most common pathological type of BLCA is urothelial carcinoma, which can be further classified into two subtypes based on the muscle invasiveness, including 75% non-muscle-invasive bladder cancers (NMIBC) and 25% muscle-invasive bladder cancers (MIBC)^2,3^. NMIBC frequently recurs (50–70%) but rarely progresses to invasion (10–50%), with a 5-year survival rate of approximately 90%^4^. Compared with NMIBC, MIBC is more aggressive and is associated with worse prognosis (< 50% for patients with localized disease and < 10% for patients with distant metastases) despite radical surgery^5^. Therefore, it’s urgently needed to develop novel therapeutic agents for these difficult-to-treat BLCA patients.

The last decade has witnessed a paradigm shift in BLCA therapy as therapeutic options for muscle-invasive and advanced BLCA expand to include cancer immunotherapy with checkpoint blockade, targeted therapy, and antibody–drug conjugates^5^. However, due to the widespread tumor heterogeneity, the responses of advanced BLCA patients to novel therapeutic agents are limited, predisposing them to inferior clinical outcomes^6^. Although intrinsic molecular subtypes (luminal/basal) have been used for prognostication and therapy response prediction, the clinical value of such subtypes in the prognosis of BLCA remains controversial^2^. Recent studies have highlighted the important roles of the molecular characteristics of patients in improving the therapeutic effects for advanced BLCA^7,8^. Therefore, there is an urgent need to develop an effective gene signature-based risk model in order to guide clinical treatments, especially for chemotherapy and immunotherapy.

Pyroptosis, a newly discovered form of programmed cell death, is initially described in myeloid cells infected by pathogens in 1992^9^. In recent years, the biggest breakthrough in this field has been the elucidation of the roles of gasdermin family as executors of pyroptosis^10,11^. Gasdermins can be cleaved by caspase-1/4/5/11 to release their N-terminal domains, which perforate cell membranes to induce membrane rupture and the release of cytosolic contents into the extracellular environment, amplifying the local or systemic inflammatory response and activating the cellular pyroptosis^10,12^. In the past decade, emerging evidence has demonstrated that pyroptosis could also play important roles in the cancer pathophysiology, regulation of tumor immune microenvironment and modulating the efficacy of immunotherapy and chemotherapy^13^. Zhang et al. found that gasdermin E (GSDME) could function as tumor suppressor gene by activating pyroptosis, which was cleaved by caspase 3 and granzyme B, enhancing macrophage-mediated anti-tumor immunity^14^. Wang et al.^15^ also used a biorthogonal system to further verify the robust pyroptosis-induced anti-tumor immunity in a mouse model, which could synergize with cancer immunotherapy using checkpoint blockade. In addition, many chemotherapeutic agents could induce caspase-3/GSDME-dependent pyroptosis in normal cells, which might explain the side effects of chemotherapy^16–18^. However, a recent study found that pyroptosis occurred in tumor cells located in the central hypoxia region, contributing to chronic tumor necrosis and a dampened anti-tumor immunity, which in turn promoted tumor progression^19^. Thus, the above studies have shown that the role of pyroptosis in anti-tumor or tumor-promoting remains controversial^20,21^. Extensive research is still needed to clarify the role of pyroptosis in the development of cancers, especially in the understudied BLCA.

In this study, we aim to build a pyroptosis-related prognostic model to predict the prognosis, evaluate the tumor infiltrating immune landscape, and inform chemotherapy and immunotherapy response in BLCA. First, 414 BLCA samples from The Cancer Genome Atlas (TCGA) database were divided into two subtypes based on pyroptosis-related genes (PRGs). A total of 953 differentially expressed genes (DEGs) between two subtypes were identified and then used to constructed a prognostic model. Univariate and multivariate COX regression as well as the least absolute shrinkage and selection operation (LASSO) were used to screen the prognosis-related DEGs, and the risk model was constructed and validated in the Gene Expression Omnibus (GEO) cohort. Finally, a nomogram which combined with clinical information and prognostic risk score was established to comprehensively predict BLCA patients’ overall survival (OS). Collectively, our study showed the prognostic value of pyrotosis-related risk model, which had the potential to predict clinical outcomes and responses to chemotherapy and immunotherapy for BLCA.

## Materials and methods

### Data collection and processing

All datasets used in this study were collected from the public available databases, including TCGA and GEO. The RNA-seq expression data and matched clinical information of 414 BLCA patients were downloaded from TCGA database (https://portal.gdc.cancer.gov/), and were used as the training dataset. GSE13507 dataset, including RNA expression data and clinical information of 246 BLCA patients, was sourced from GEO database (https://www.ncbi.nlm.nih.gov/geo/), and was used as external validation dataset. The RNA expression data of GSE13507 dataset, generated by Illumina human-6 v2.0 expression beadchip, was converted to transcripts per kilobase million (TPM) format using R package limma for downstream analyses^22^. 57 pyroptosis-related genes (PRGs) were obtained from REACTOME database (https://reactome.org/), Gene Ontology (GO) database (http://geneontology.org), and published articles^23^ (Table S1). The whole analysis pipeline was shown in the flow chart (Figure S1).

### Identification of survival-related differentially expressed PRGs in BLCA

DESeq2 package in R software was utilized to screen the differentially expressed genes (DEGs) between 414 BLCA and 19 normal bladder mucosa samples in the training dataset with the criteria of |log_2_ (Fold change (FC))|> 1 and adjusted *P* value < 0.05^24^. Then, the differentially expressed PRGs (DEPRGs) were obtained by interesting DEGs with 57 PRGs. Next, BLCA patients in the training dataset were divided into low- and high-expression groups according to the median expression level of each DEPRG. The survival between low- and high-expression group was analyzed and compared by Kaplan–Meier curves and log-rank test analysis. DEPRGs were considered to be associated with the overall survival of BLCA patients when there was significant survival difference between low- and high-expression groups (*P* value < 0.05).

### Consensus clustering of BLCA patients based on survival-related DEPRGs

Consensus clustering was performed by ConsensusClusterPlus package to divide the BLCA patients into distinct pyroptosis-related subgroups based on the expression of survival-related DEPRGs^25^. The number of the clusters were determined by k value, and the stability of classification was guaranteed by performing 1,000 times repetitions. t-distributed stochastic neighbor embedding (t-SNE) was further conducted to assess the performance of consensus clustering. Furthermore, to characterize the subgroups, we (1) analyzed and compared the survival of patients in different subgroups by Kaplan–Meier curves and log-rank test, (2) analyzed and compared the distribution of clinical features (age, gender and TNM stage) in different groups, (3) performed the gene set enrichment analysis (GSEA) with R package GSVA to explore the significantly enriched biological processes and pathways between different groups using gene ontology (GO) and Kyoto Encyclopedia of Genes and Genomes (KEGG)^26^ reference gene sets downloaded from MSigDB database^27^ (https://www.gsea-msigdb.org/gsea/msigdb/), (4) evaluated and compared the proportions of immune cells in the tumor microenvironment (TME) between subgroups using the CIBERSORT algorithm^28^, immune/stromal/ESTIMATE score via the ESTIMATE algorithm^29^ and expression levels of immune checkpoint genes (ICGs).

### Development and validation of pyroptosis-related risk gene signature

DEGs were screened between pyroptosis-related subclusters using DESeq2 package with |log_2_FC|> 1 and adjusted *P* value < 0.05. Thereafter, univariate Cox and LASSO regression analyses were performed to select genes related to the survival of BLCA. Then, these pyroptosis-related DEGs were loaded into multivariate Cox proportional hazards model to construct the risk gene signature, and the risk score of each BLCA patient in TCGA cohort was calculated as follows: Risk score = $\sum\nolimits_{i = 1}^{n} {\left[ {coefficient \left( {Genei} \right)*expression \left( {Genei} \right)} \right]}$. BLCA patients in the training dataset were divided into low- and high-risk groups based on the median of risk score. Kaplan–Meier curves and log-rank test were used to analyze and compare the survival difference between low- and high-risk groups (*P* value < 0.05). The 1-, 3-, and 5-year time-dependent receiver operator characteristic curve (ROC) curves were plotted by R package survivalROC to evaluate the performance of the risk signature^30^. Moreover, the risk score model was validated in the external GSE13507 dataset. In addition, multivariate Cox regression analysis was performed to identify independent prognostic factors, followed by the establishment of a nomogram using R package rms for the prediction of 1-, 3- and 5-year survival of BLCA patients. The performance of the nomogram was evaluated by the calibration curves.

### Analysis of immunotherapeutic response and chemotherapeutic sensitivity

Tumor Immune Dysfunction and Exclusion (TIDE) score of each sample in high- and low-risk groups was calculated using TIDE database (http://tide.dfci.harvard.edu/)^31^. Difference of TIDE prediction score between high- and low-risk groups was analyzed by Wilcoxon rank sum test. High TIDE score indicated poor outcome of immune checkpoint blockade (ICB) and short survival time after treatment. In addition, based on Genomics of Drug Sensitivity in Cancer (GDSC) online database, the half maximal inhibitory concentration (IC_50_) value of cisplatin for each patient was calculated^32^, and the differences between high- and low-risk groups were also determined by Wilcoxon rank sum test.

### Statistical analysis

Continuous variables were compared between two subgroups using Student’s t test for normally distributed variables, and Wilcoxon rank sum test was used to estimate the statistical significance of non-normally distributed variables. Fisher’s test or Chi-square test was used for categorical variables. The Kaplan–Meier method with a two-sided log rank test was used to compare the OS of patients between subgroups. Univariate and multivariate Cox proportional hazards regression models were used to assess the independent prognostic value of the risk model. Time-dependent ROC curve analyses were used to estimate the accuracy of the predictive and prognostic value of the risk gene signature model. All statistical analyses were performed with R software (v4.1.0). *P* value less than 0.05 was considered to be statistically significant.

## Results

### Pyroptosis-related GSDMA and CHMP4C were related to the development and progression of BLCA

**Figure 1:**
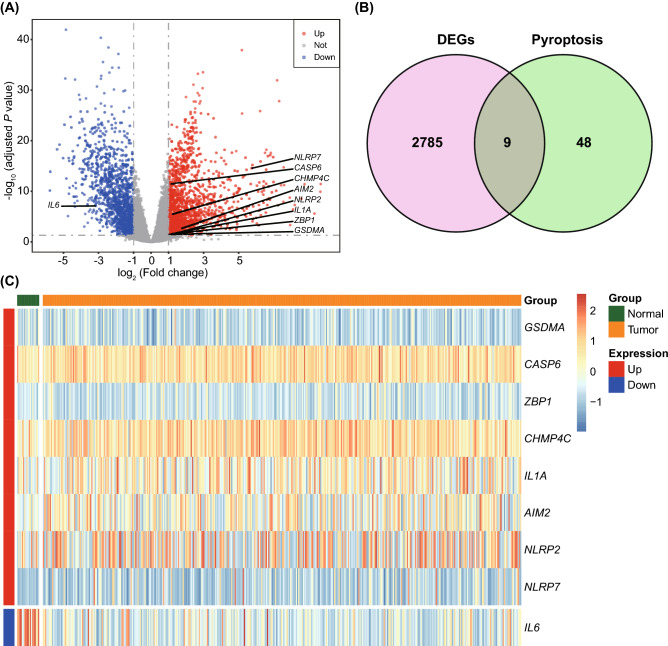
Identification of the differentially expressed PRGs. (**A**) Volcano plot showing the DEGs between normal and tumor tissues; (**B**) Venn plot showing the intersection between 2794 DEGs and 57 pyroptosis genes; (**C**) Heatmap of nine DEPRGs. PRGs, pyroptosis-related genes; DEGs, differentially expressed genes; DEPRGs, differentially expressed pyroptosis-related genes.

**Figure 2:**
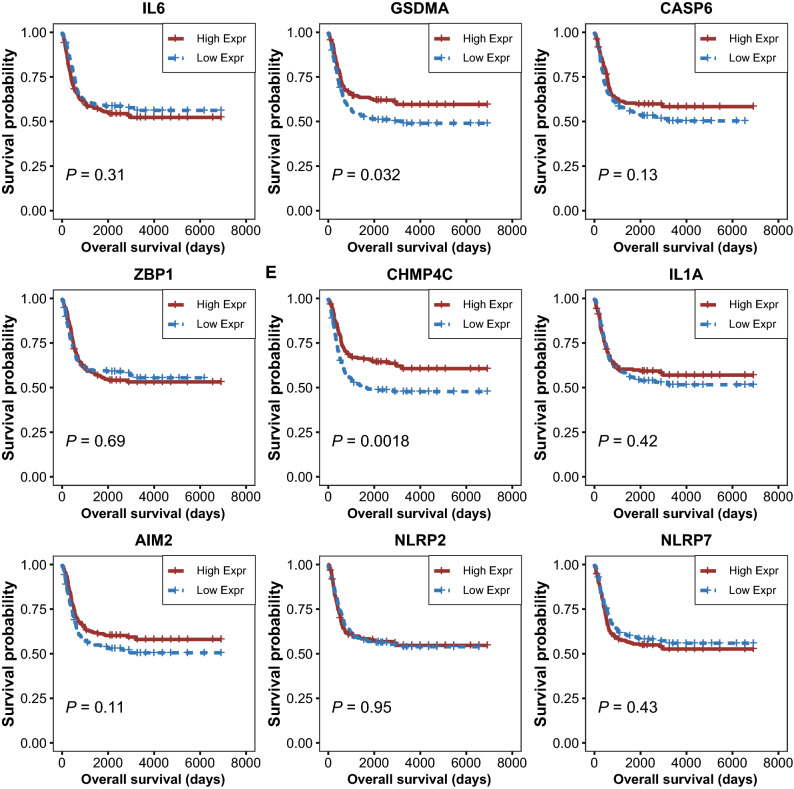
Kaplan–Meier curves showing the overall survival of patients with high or low expression of nine DEPRGs.

**Figure 3:**
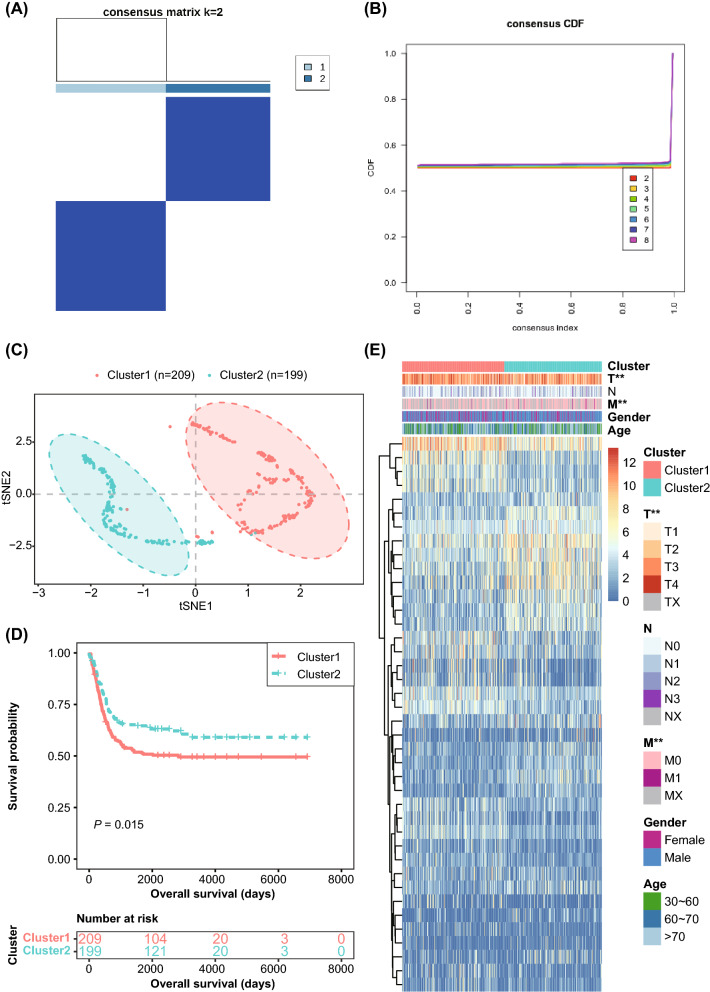
Identification of subtypes of BLCA based on two DEPRGs associated with patients’ survival. (**A**) BLCA patients were grouped into two distinct subtypes using unsupervised clustering method (k = 2); (**B**) Empirical CDF plot displaying consensus distribution for each k; (**C**) t-SNE plot for validation of the stability and reliability of the two distinct subtypes; (**D**) Kaplan–Meier curves showing the significant different OS for BLCA patients between cluster 1 and 2 subtypes; (**E**) Heatmap showing the clinicopathological characteristics of the two clusters stratified by DEPRGs expression patterns. BLCA, bladder cancer; CDF, cumulative distribution function; t-SNE, t-distributed stochastic neighbor embedding; OS, overall survival.

A total of 2,794 DEGs, including 1,509 upregulated and 1,285 downregulated genes, were identified between BLCA and normal tissues (Table S2 and Fig. 1A). By intersecting 2,794 DEGs with 57 PRGs, nine DEPRGs, including *IL6*, *GSDMA*, *CASP6*, *ZBP1*, *CHMP4C*, *IL1A*, *AIM2*, *NLRP2*, and *NLRP7*, were obtained (Fig. 1B). The expression levels of DEPRGs were displayed in the heatmap, indicating that *GSDMA*, *CASP6*, *ZBP1*, *CHMP4C*, *IL1A*, *AIM2*, *NLRP2*, and *NLRP7* were significantly elevated, whereas the expression of *IL6* was markedly reduced in BLCA samples compared with those in controls (Fig. 1C). Further Kaplan–Meier analysis showed that BLCA patients with higher expression of *GSDMA* and *CHMP4C* had better overall survival (Fig. 2), indicating that pyroptosis-related GSDMA and CHMP4C had close relationship with BLCA survival. To further explore the expression patterns of PRGs in BLCA, a consensus clustering algorithm was used to categorize the BLCA patients based on the expression patterns of *GSDMA* and *CHMP4C*. Our results showed that k = 2 was the optimal parameter for classifying the entire TCGA training set into cluster 1 (n = 209) and cluster 2 (n = 199) (Fig. 3A,B). The t-SNE analysis showed the distinct differences of samples between these two clusters (Fig. 3C), further demonstrating the reliability of consensus clustering. Interestingly, we observed a better survival of patients in cluster 2 (log-rank test, *P* = 0.015, Fig. 3D). Furthermore, the comparisons of clinicopathological features between these two clusters revealed that the distributions of patients at T and M stage were significantly different (Fig. 3E), suggesting that pyroptosis-related GSDMA and CHMP4C had close relationship with the development and progression of BLCA.

### Two subtypes had distinct characteristics of tumor microenvironment

**Figure 4:**
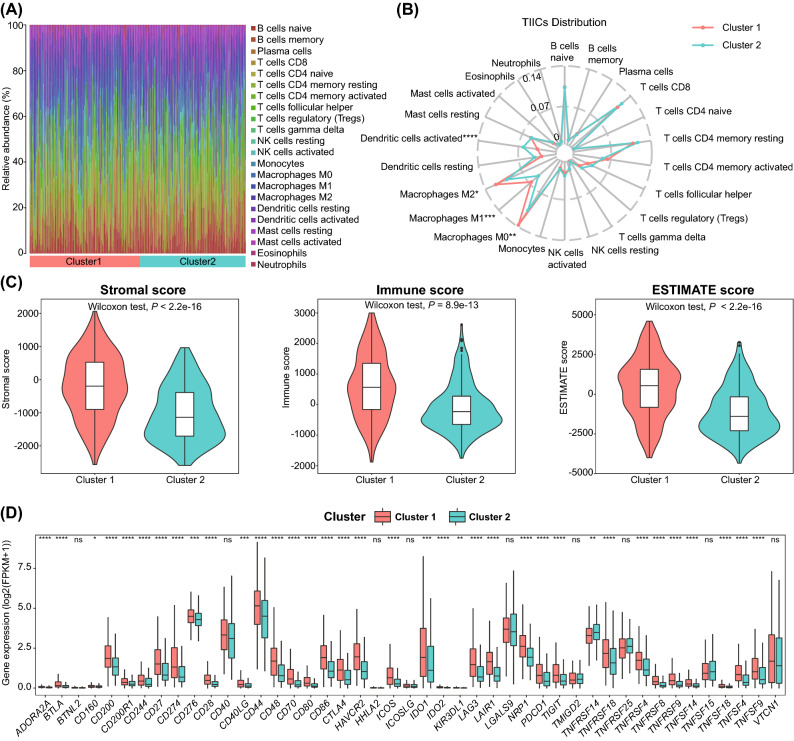
Association of tumor immune microenvironment between two pyroptosis-related subtypes. (**A**) Barplot showing the composition of 22 immune cells in two clusters; (**B**) Radar chart displaying the TIICs distribution of cluster 1 (Red) and 2 (Green); (**C**) Correlations of the TME score and the two BLCA subtypes; (**D**) Boxplot showing the gene expression differences of ICGs between two pyroptosis-related clusters. TIICs, tumor infiltrating immune cells; TME, tumor microenvironment; ICGs, immune checkpoint genes.

Numerous reports have shown the close relationship between pyroptosis and TME^33–35^. Thus, we further investigated the characteristics of TME in the two pyroptosis-related clusters. We first analyzed the immune landscape (22 human immune cells) of the two clusters using the CIBERSORT algorithm (Fig. 4A), and found that the infiltration levels of activated dendritic cells (DCs) were significantly higher in cluster 2 than those in cluster 1, while M0/M1/M2 macrophages had significantly higher infiltration in cluster 1 compared to cluster 2 (Fig. 4B). Moreover, we also calculated the TME score (stromal score, immune score, and ESTIMATE score) of the two clusters using the ESTIMATE algorithm. The results demonstrated that cluster 1 had markedly higher immune, stromal, and ESTIMATE scores than cluster 2 (Fig. 4C). In addition, the expression levels of 45 ICGs were also significantly different between these two clusters, which had much higher expression levels in the majority of ICGs in cluster 1 compared with those in cluster 2 (Fig. 4D). These above results indicated that clustering subtypes based on the gene expression profiles of pyroptosis-related GSDMA and CHMP4C were closely associated with TME in BLCA.

### Identification of DEGs between pyroptosis-related clusters and functional enrichment analysis

**Figure 5:**
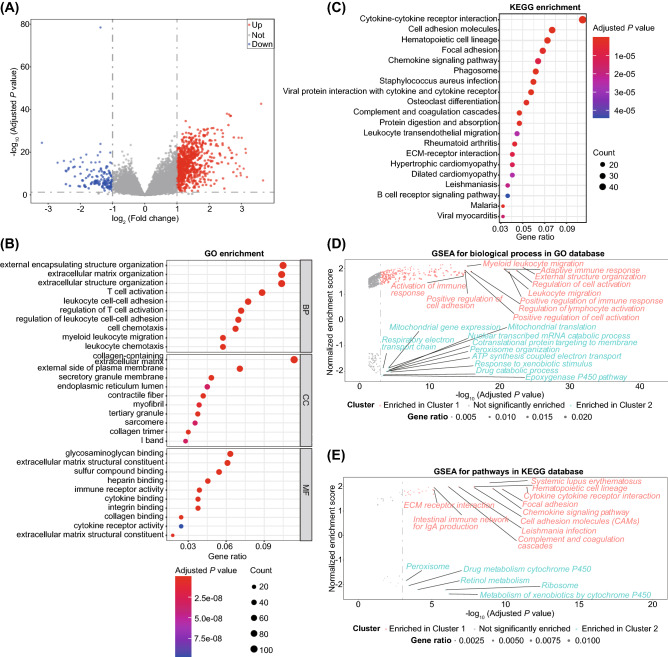
Function enrichment analysis of DEGs between two pyroptosis-related subtypes. (**A**) Volcano plot showing the DEGs between two subtypes; (**B**) GO enrichment analysis (BP, CC, and MF) of DEGs between two pyroptosis-related subtypes; (**C**) KEGG enrichment analysis of DEGs; (**D**) GSEA enrichment analysis for GO BP database; (**E**) GSEA enrichment analysis for KEGG database^26^. DEGs, differentially expressed genes; GO, gene ontology; BP, biological process; CC, cellular component; MF, molecular function; KEGG, Kyoto encyclopedia of genes and genomes (www.kegg.jp); GSEA, gene set enrichment analysis.

TO explore the potential biological functions of the two pyroptosis patterns, we identified a total of 953 pyroptosis-related DEGs using the R package “DESeq2” (Table S3 and Fig. 5A) and performed functional enrichment analysis. These DEGs were significantly enriched in 1049 biological processes (BPs), 84 cellular components (CCs), 87 molecular functions (MFs) and 275 KEGG pathways (TableS S4 and S5). These DEGs were mainly involved in extracellular matrix (ECM) and immunity-related GO terms, such as BPs of ECM organization, T cell activation, and myeloid leukocyte migration, CCs of ECM, secretory granule membrane, and MFs of ECM structural constituent, immune receptor activity, cytokine binding, and cytokine receptor activity (Fig. 5B). KEGG analysis indicated the enrichment of immune-related pathways, such as cytokine-cytokine receptor interaction, cell adhesion molecules, focal adhesion, chemokine signaling pathway, and complement and coagulation cascades (Fig. 5C). Moreover, GSEA also found that ECM and immunity-related BPs and KEGG pathways were mainly enriched in cluster 1, such as BPs of adaptive immune response, positive regulation of cell adhesion (Fig. 5D), and KEGG pathways of cytokine-cytokine receptor interaction, ECM receptor interaction (Fig. 5E), demonstrating the important roles of pyroptosis in ECM organization and immune regulation in BLCA.

### Construction and validation of risk gene signature based on DEGs between two pyroptosis-related clusters

**Figure 6:**
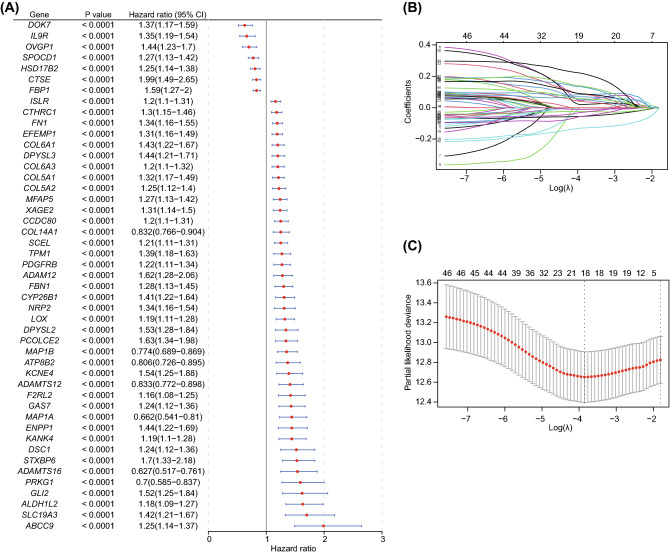
Screening of prognosis-associated DEGs between two subtypes related to pyroptosis. (**A**) Forest plot showing the prognostic genes based on DEGs between two pyroptosis-related subtypes by univariate Cox regression analysis; (**B**) Profiles of LASSO regression coefficient for 47 DEGs; (**C**) Identification of tuning parameter (λ) in the LASSO Cox model by ten-fold cross-validation. DEGs, differentially expressed genes; LASSO, least absolute shrinkage and selection operator.

To better predict the survival of BLCA patients, we then established an accurate prognostic risk gene signature based on the pyroptosis-related DEGs. Firstly, all BLCA patients in the TCGA cohort were selected as the training dataset (n = 405). 47 DEGs between the two clusters were found to be associated with the prognosis of BLCA by univariate Cox regression analysis (*P* < 0.0001, Fig. 6A). Then, 18 genes were further selected as prognostic signature genes at lamda.min of 0.021 by LASSO regression analysis (Fig. 6B,C). Finally, 15 genes (Cox proportional hazards test, *P* < 0.05), including ABCC9, COL14A1, CTSE, PCOLCE2, STXBP6, SPOCD1, FBP1, IL9R, KANK4, XAGE2, DOK7, OVGP1, DSC1, F2RL2, and SCEL, were included into multivariate Cox analysis to construct the risk gene signature model in the training dataset. The risk score was calculated as follows:

Risk score = (0.06268*expression of ABCC9) + (0.11150*expression of COL14A1) + (− 0.06791*expression of CTSE) + (0.04706*expression of PCOLCE2) + (0.09674*expression of STXBP6) + (− 0.02104*expression of SPOCD1) + (− 0.01833*expression of FBP1) + (− 0.09528*expression of IL9R) + (0.13209*expression of KANK4) + (0.06433*expression of XAGE2) + (− 0.14266*expression of DOK7) + (− 0.07570*expression of OVGP1) + (0.25217*expression of DSC1) + (0.17517*expression of F2RL2) + (0.06990*expression of SCEL).

**Figure 7:**
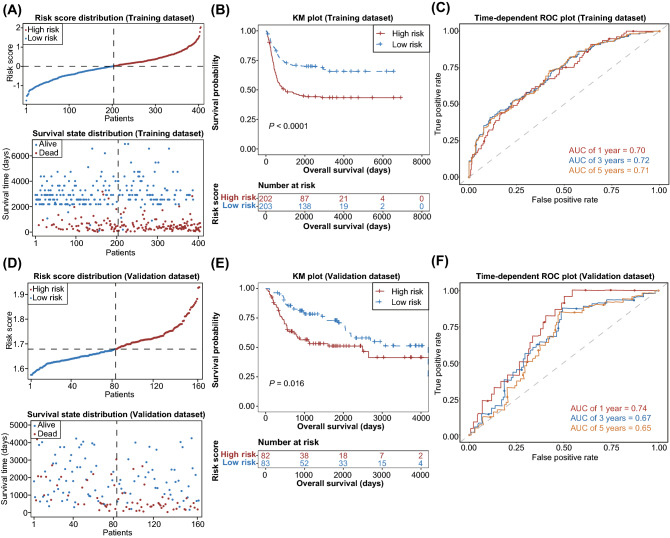
Construction of prognostic signature based on prognosis-related DEGs between two proptosis-related subtypes. (**A**) Distribution of risk score (up panel) and survival status (down panel) in the training dataset (TCGA cohort); (**B**) Kaplan–Meier curves showing the overall survival of BLCA patients in high- and low-risk groups in training dataset; (**C**) Time-dependent ROC curves demonstrated the predictive efficiency of the risk model in the training dataset; (**D**) Distribution of risk score (up panel) and survival status (down panel) in the validation dataset (GEO cohort); (**E**) Kaplan–Meier curves showing the overall survival of BLCA patients in high- and low-risk groups in validation dataset; (**F**) Time-dependent ROC curves demonstrated the predictive efficiency of the risk model in the validation dataset. TCGA, the cancer genome atlas; BLCA, bladder cancer; ROC, receiver operating characteristics; GEO, gene expression omnibus.

**Figure 8:**
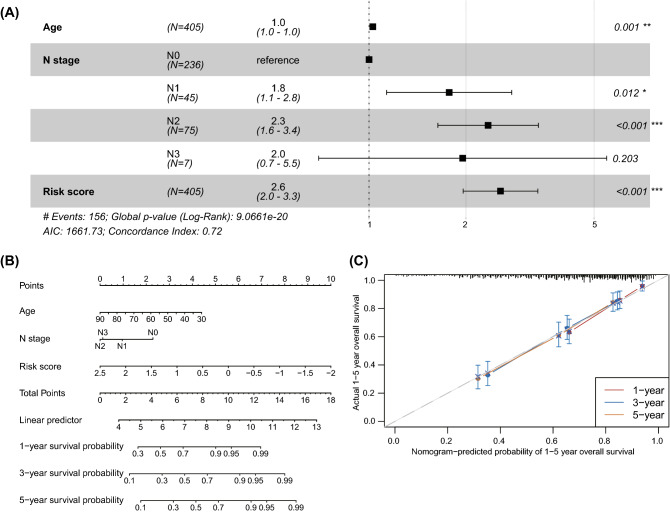
Development and validation of a nomogram for survival prediction. (**A**) Forest plot showed the independent factors including clinical traits and risk score using univariate and multivariate Cox regression analyses; (**B**) A nomogram based on age, N stage, and risk score was constructed to predict the 1-, 3-, and 5-year overall survival; (**C**) Calibration curves showed the similar performance of the nomogram model compared to the ideal model.

According to the median value of risk score, BLCA patients were divided into low- and high-risk groups (Fig. 7A). We found that patients in low-risk group had better survival than those in the high-risk group (Fig. 7B). To evaluate the performance of the risk model, ROC curves were plotted and the AUCs for 1-, 3-, 5-year were 0.70, 0.72 and 0.71, respectively (Fig. 7C), indicating the high predictive accuracy of the risk model. Moreover, the risk model was validated in an external GSE13507 dataset (n = 165), and similar results were obtained (Fig. 7D–F), suggesting the stability and reliability of this risk model. In addition, we found that the risk score, age and N stage were independent prognostic factors in BLCA by multivariate regression analysis (Fig. 8A). To make it easier for clinicians to predict the survival probability of BLCA patients, a nomogram model was constructed based on risk score, age and N stage (Fig. 8B). The calibration curve was plotted to evaluate the nomogram’s performance. Our data showed that the predicted probability was close to the actual survival (Fig. 8C), indicating the clinical utility value of this nomogram in BLCA.

### The pyroptosis-related risk score was associated with immune—and chemotherapeutic efficacy

**Figure 9:**
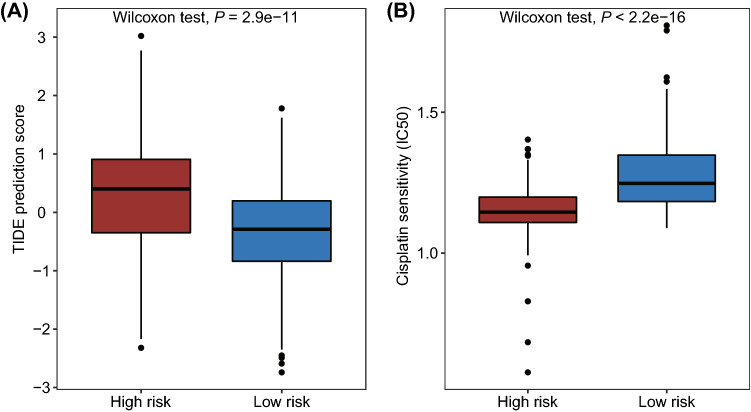
The role of pyroptosis-related risk score in the prediction of therapeutic response. (**A**) The correlation between TIDE score and risk score; (**B**) Boxplot showed the relationship between IC_50_ value of cisplatin and risk score.

Previous studies have shown that the molecular subtype of BLCA can predict its clinical response to immune checkpoint blockade therapy and the neoadjuvant chemotherapy^2,4^. To further explore the potential capability of pyroptosis-related risk score, we firstly investigated the relationship between the risk score and immunotherapy efficacy. Our results showed that patients in low-risk group had lower TIDE score (Fig. 9A), indicating the better immunotherapy response for low-risk patients. As for chemotherapy, we focused on the small molecule compound, cisplatin, which was used as the first-line chemotherapy for BLCA. We found that the IC_50_ of cisplatin were much higher in low-risk group (Fig. 9B), suggesting that patients in high-risk group were more sensitive to cisplatin treatment. Collectively, these results showed that our risk signature was associated with therapeutic efficacy of BLCA patients, and immunotherapy and neoadjuvant chemotherapy may be used, either alone or in combination, in the treatment of BLCA patients with high pyroptosis-related risk score.

## Discussion

Due to the high recurrence rate, metastasis rate, and limited therapeutic agents, advanced BLCA remains a serious tumor disease of the urinary system in clinical practice^1,5^. Therefore, it is meaningful to develop novel diagnostic biomarkers, therapeutic targets, and risk model to predict patients’ prognosis and guide chemotherapy and immunotherapy for BLCA. Numerous studies have highlighted the critical role of pyroptosis in modulating innate immunity and ani-tumor effects^13,20,21^. However, the specific molecular mechanism of pyroptosis remains to be fully elucidated in BLCA. Here, we integrated transcriptome data from TCGA and GEO database to comprehensively characterize pyroptosis-related clinical features and gene signature in BLCA. We identified two distinct subtypes based on two DEPRGs, namely, *GSDMA* and *CHMP4C*. Compared with patients in cluster 2, patients in cluster 1 had more serious clinicopathological features and worse OS. These two subtypes also displayed distinct characteristics of the TME. Furthermore, we identified 953 pyroptosis-related DEGs between two different subtypes, which significantly enriched ECM and immunity-related pathways. Based on these DEGs of the pyroptosis subtypes, we established a risk model to predict the prognosis for BLCA patients. By integrating the pyroptosis-related risk score and clinical variables, we constructed a nomogram to predict the overall survival of BLCA patients. Finally, we found that high risk score was significantly associated with high TIDE score and low IC_50_ of cisplatin, demonstrating the clinical value of the prognostic model in predicting immunotherapeutic and chemotherapeutic efficacy. Our study not only provides clinicians with a practical prognostic model for stratification of patients, but will also further enhance our understanding of the molecular mechanism of pyroptosis in BLCA.

Interestingly, while our manuscript was under review, several studies using similar strategy to develop PRGs-based risk models were published^36–38^. We performed a comprehensive analysis of every relevant aspect of the risk model, including the pyroptosis-related gene sets, training and validation datasets, model construction methods and performance (Table S6). Overall, our risk model had better performance in predicting BLCA patients’ prognosis. Moreover, these risk models had some slight differences in the number of PRGs, the strategy of model construction, and the training and validation datasets used. For example, Zhang et al.^38^ used the gene expression patterns of the entire PRG gene set to conduct consensus clustering, while Chen et al.^36^, Deng et al.^37^ and our study used DEPRGs. In our study, we initially used all PRGs to classify BLCA patients. All patients could be divided into two clusters (Figure S2A, B). However, patients could not be clearly distinguished between the two clusters in the tSNE plot (Figure S2C), and the overall survival of the two subtypes showed no significant difference (*P* = 0.44, Figure S2D). Therefore, we adopted the survival-associated DEPRGs to conduct the subtype analyses.

Our study identified significantly upregulated pyroptosis-related genes *GSDMA* and *CHMP4C* as favorable prognostic factors in BLCA patients. To date, the molecular functions of these two DEPRGs have not been well characterized in BLCA. GSDMA is one of the gasdermin family with pore-forming activity that functions as the key executioners of pyroptosis, which is mostly expressed in the epithelial cells of the skin, esophagus, and bladder and is silenced in the gastric cancer cell lines and tissues^11^. These evidences implied that *GSDMA* might be a tumor suppressor gene in gastric cancers. Saeki et al. also showed that restoration of GSDMA in human gastric cancer cell lines could increase their sensitivity to cellular apoptosis^39^. On the contrary, Orita et al. found that GSDMA was not expressed in normal colorectal tissues but was gradually overexpressed in matched cancer tissues, suggesting that it might act as tumor promotor gene in colorectal cancer^40^. A recent study showed that exotoxin B produced by *Streptococcus pyogenes* could cleave GSDMA and trigger keratinocyte pyroptosis, which verified the speculation that it acted as the host-defense protein in skin^41,42^. CHMP4C is an important member of the chromatin-modifying protein/charged multivesicular body protein (CHMP) family. These proteins are parts of endosomal sorting complex required for transport III (ESCRT-III), which is involved in degradation of surface receptor proteins and formation of endocytic multivesicular bodies (MVBs)^43,44^. During pyroptosis, ESCRT-mediated cell membrane repair can negatively regulate pyroptosis triggered by the activation of GSDMD^45^. Chen et al. found that silencing of *CHMP4C* could obviously inhibit the proliferation, migration, and invasion of pancreatic adenocarcinoma cells^46^. Lin et al. also discovered that CHMP4C accelerated the cell proliferation and migration via activation of epithelial-mesenchymal transition pathway in cervical cancer^47^. These findings were not consistent with our data, in which CHMP4C acted as a tumor suppressor gene. More studies are needed to elucidate the reasons for the differences in the roles of CHMP4C across different cancers in the future.

In the past decade, breakthroughs have been made in the treatment of BLCA with immunotherapy. However, the heterogeneity of anti-tumor immunity is strongly linked cancer progression and response to immunotherapy^48^. TME is composed of tumor infiltrating immune cells (TIICs), blood vessels, fibroblasts, and ECM. Emerging evidence has also shown the significant roles of pyroptosis in modulating the TME, thereby affecting tumor development, progression, and therapeutic resistance^49^. In the present study, the pyroptosis subtype characterized by immune activation (cluster 2) was associated with a better prognosis. Immune checkpoint genes associated with immunosuppression, such as CD274, CTLA4, IDO1, and LGALS9, were markedly lower in patients in cluster 2. In addition, patients in cluster 2 had lower stromal/immune/ESTIMATE scores, which was consistent with the better prognosis. Accordingly, we also discovered that the relative abundance of 22 TIICs was significantly different between two pyroptosis-related subtypes, implying the key role of pyroptosis in shaping TME. Interestingly, we found that myeloid cells, including macrophage M0/M1/M2 and activated DCs, showed significant differences in the two subtypes. Cluster 2 with better prognosis showed higher infiltration of activated DCs, suggesting that they play a positive role in BLCA. Although the fraction of DCs in the TME was small, they had the potent capability to foster T cell immunity and enhance immunotherapy responses^50^. Tumor-associated macrophages are polarized into two main phenotypes, namely M1 macrophages and M2 macrophages, which inhibit or promote cancer progression, respectively. In this study, the fraction of M2 macrophages with immunosuppressive phenotypes in TME was significantly lower in cluster 2, which was associated with the favorable prognosis. Intriguingly, we also found decreased infiltration of M1 macrophages in cluster 2. We speculated that this might be related to the stronger plasticity of macrophages^51–53^. To improve the accuracy of dissecting the TME composition, sing-cell transcriptome datasets might be required to analyze the proportion of macrophage under different polarized states in the future.

We further explored the relevant signaling pathways and functions of DEGs between different pyroptosis-related subtypes. Both KEGG and GO enrichment analysis indicated that DEGs significantly enrich a variety of biological functions related to ECM and immunity, including ECM organization, T cell activation, myeloid leukocyte migration, and cytokine-cytokine receptor interaction. These findings were consistent with the estimated stromal and immune score by ESTIMATE algorithm. In addition, we have also established a nomogram for predicting prognosis at 1, 3, and 5 years, which can help clinicians to accurate stratification of BLCA patients. Last but not least, we found that this prognostic model could guide immunotherapy and chemotherapy, which would bring clinical benefits to BLCA patients.

There were some limitations for this study. Firstly, our findings were solely based on public datasets that were composed of retrospective data. Large-scale prospective clinical cohort validation in the real world is needed. Secondly, we used a relatively small number of paracancerous tissues (n = 19) in the TCGA-BLCA dataset as normal controls to identify DEGs compared to the BLCA tissues (n = 414), which may cause bias. In addition, paracancerous tissues, the tissue adjacent to a solid tumor, although histologically normal, are not normal tissues after all, which might suffer from a field effect. Thirdly, the molecular mechanism of pyroptosis shaping TME characteristics in BLCA was still unknown. Additional in vivo and in vitro functional studies should be investigated experimentally in the future.

In summary, we utilized a comprehensive bioinformatics approach to dissect the molecular mechanism of pyroptosis in affecting TME composition, clinicopathological features, and patients’ prognosis in BLCA. We also constructed a pyroptosis-related prognostic model to inform immunotherapy and chemotherapy. Our findings not only provided novel insights into the role of pyroptosis in BLCA, but also supplied potential independent prognostic factors for diagnosis and therapy of patients with BLCA.

## Supplementary Information

Supplementary Information 1.

Supplementary Information 2.

Supplementary Information 3.

Supplementary Information 4.

Supplementary Information 5.

Supplementary Information 6.

Supplementary Information 7.

Supplementary Information 8.

Supplementary Information 9.

## Data Availability

The datasets analyzed during the current study are available in the TCGA database (https://portal.gdc.cancer.gov) and GEO database (https://www.ncbi.nlm.nih.gov/geo/query/acc.cgi?acc=GSE13507). The code and related datasets used in the overall bioinformatic analyses are available on the Github (https://github.com/tielin053/BLCA_Pyroptosis2022). All additional information required to reproduce our paper is available from the corresponding author upon request.
