# Supplementary figures and images for: A risk model based on pyroptosis subtypes predicts tumor immune microenvironment and guides chemotherapy and immunotherapy in bladder cancer
Source: Sci Rep. 2022 Dec 12;12:21467. doi: 10.1038/s41598-022-26110-4 (PMC9744904; doi:10.1038/s41598-022-26110-4)

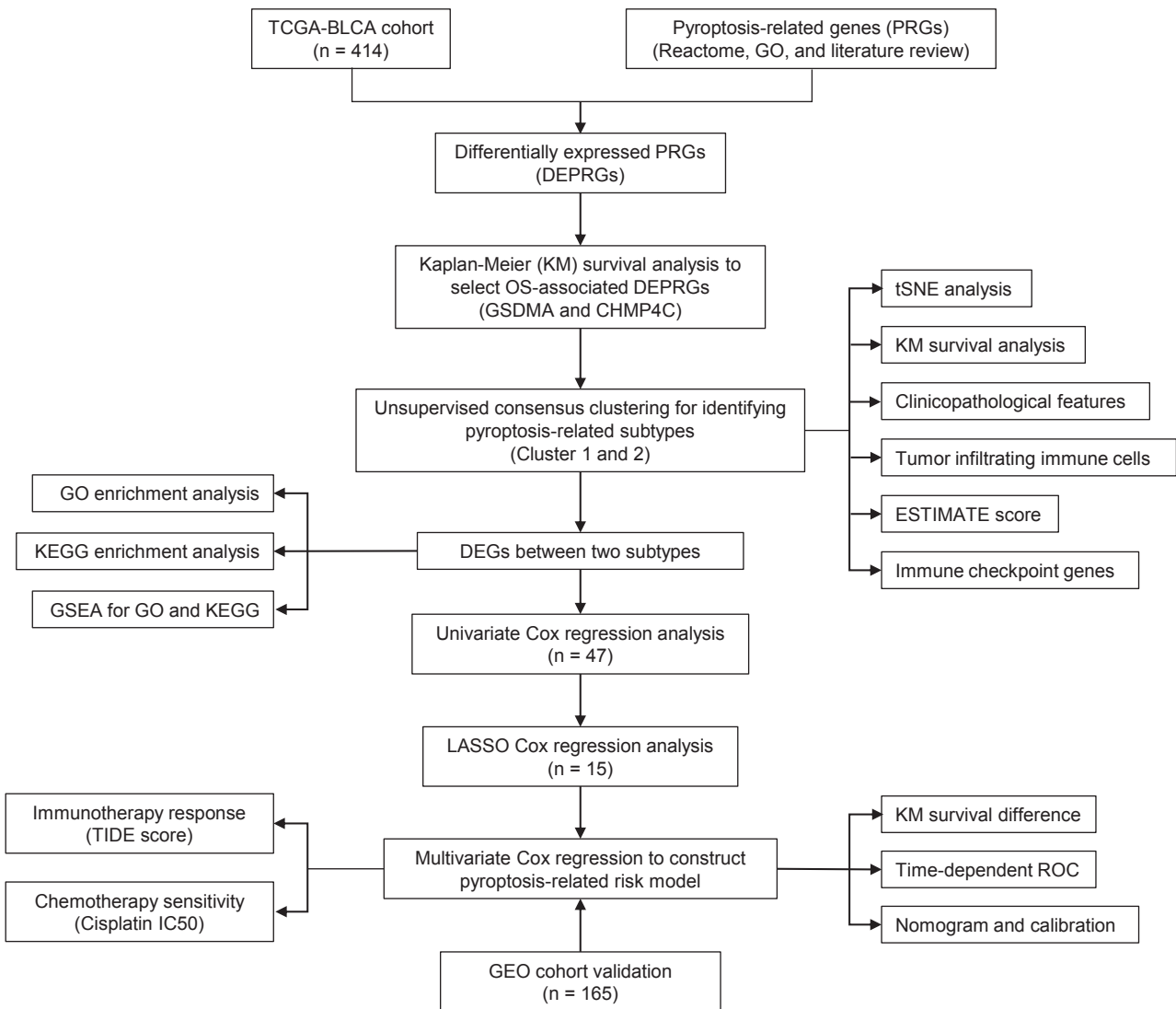

Supplement: Supplementary file 1 — Supplementary Information 1. [file 41598_2022_26110_MOESM1_ESM.pdf]

# Figure S2

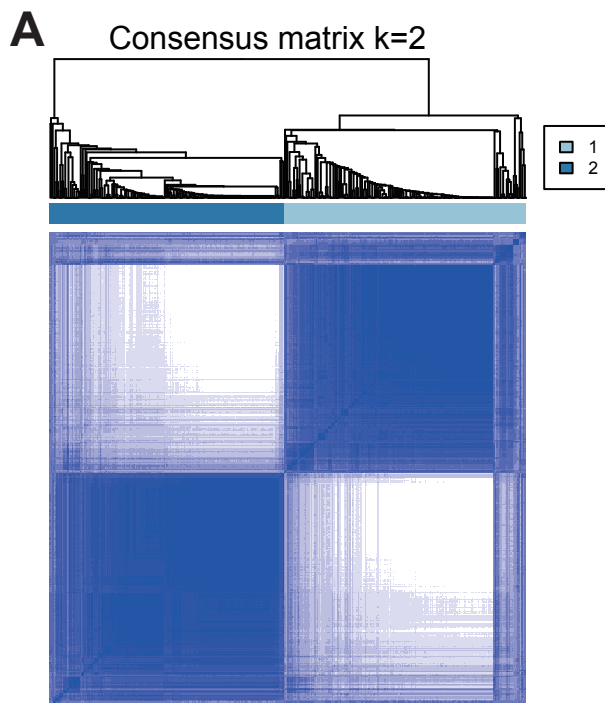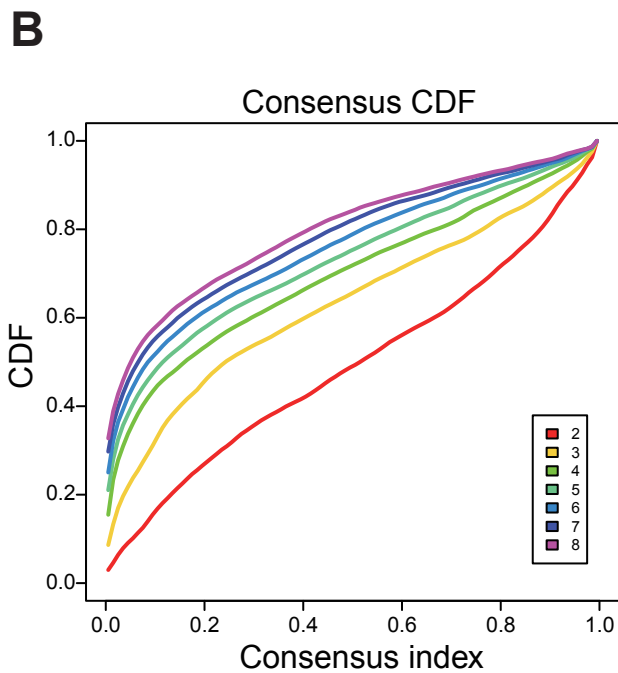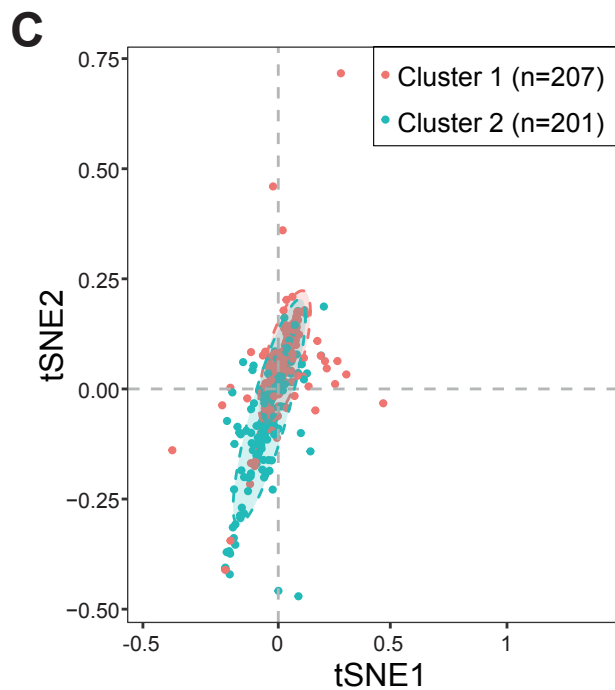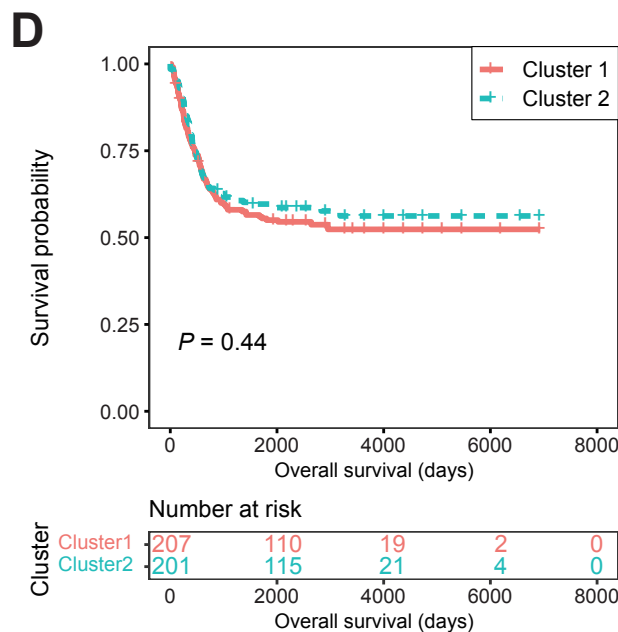

Supplement: Supplementary file 2 — Supplementary Information 2. [file 41598_2022_26110_MOESM2_ESM.pdf]
