# Supplementary material for: A risk model based on pyroptosis subtypes predicts tumor immune microenvironment and guides chemotherapy and immunotherapy in bladder cancer
Source: Sci Rep. 2022 Dec 12;12:21467. doi: 10.1038/s41598-022-26110-4 (PMC9744904; doi:10.1038/s41598-022-26110-4)
Supplement: Supplementary file 3 — Supplementary Information 3. [file 41598_2022_26110_MOESM3_ESM.docx]

**Supplementary figures**

**Figure S1 The flowchart of this study.**

**Figure S2 Identification of subtypes of BLCA based on all PRGs.**

(A) BLCA patients were grouped into two distinct subtypes using unsupervised clustering method (k = 2); (B) Empirical CDF plot displaying consensus distribution for each k; (C) t-SNE plot for validation of the stability and reliability of the two distinct subtypes; (D) Kaplan-Meier curves showing the OS for BLCA patients between cluster 1 and 2 subtypes.

**Supplementary tables**

**Table S1 Gene list of pyroptosis-related genes**

**Table S2 Identification of differentially expressed genes between BLCA and normal tissues**

**Table S3 DEGs list between two pyroptosis-related subtypes**

**Table S4 Gene ontology enrichment analysis**

**Table S5 KEGG enrichment analysis**

**Table S6 Comparison of the risk model constructed in this study with previously published reports**
